# Intestinal Fluid and Glucose Transport in Wistar Rats following Chronic Consumption of Fresh or Oxidised Palm Oil Diet

**DOI:** 10.5402/2011/972838

**Published:** 2010-10-26

**Authors:** Agona O. Obembe, Daniel U. Owu, Obem O. Okwari, Atim B. Antai, Eme E. Osim

**Affiliations:** Department of Physiology, College of Medical Sciences, University of Calabar, 540001 Calabar, Nigeria

## Abstract

Chronic ingestion of thermoxidized palm oil causes functional derangement of various tissues. This study was therefore carried out to determine the effect of chronic ingestion of thermoxidized and fresh palm oil diets on intestinal fluid and glucose absorption in rats using the everted sac technique. Thirty Wistar rats were divided into three groups of 10 rats per group. The first group was the control and was fed on normal rat chow while the second (FPO) and third groups (TPO) were fed diet containing either fresh or thermoxidized palm oil (15% wt/wt) for 14 weeks. Villus height and crypt depth were measured. The gut fluid uptake and gut glucose uptake were significantly (*P* < .001) lower in the TPO group than those in the FPO and control groups, respectively. The villus height in the TPO was significantly (*P* < .01) lower than that in FPO and control. The villus depth in TPO was significantly (*P* < .05) higher than that in FPO and control groups, respectively. These results suggest that ingestion of thermoxidized palm oil and not fresh palm oil may lead to distortion in villus morphology with a concomitant malabsorption of fluid and glucose in rats due to its harmful free radicals.

## 1. Introduction

Lipids represent a class of dietary oxidants of major nutritional and toxicological importance. These potentially toxic oxidants result from peroxidation of polysaturated fatty acids of dietary lipids. They are among the natural mutagens and carcinogens present in the human diets that can initiate degenerative processes through the generation of oxygen radicals which may ultimately lead to the damage of the digestive system [1], including intestinal inflammation and cancer [2]. 

There is also a casual relationship between toxicity of dietary polysaturated oil and the peroxide content in rats suggesting a potential cytotoxic effect associated with excessive consumption of thermoxidized palm oil with a high level of oxidation. For instance, several reports have been documented on the effect of thermally oxidized palm oil diets on various functional and structural alterations [3–5]. Furthermore, degeneration of the mucosa and submucosa reduced size of the villi which led to a general distortion in the intestinal morphology following chronic ingestion of thermoxidized palm oil have been reported [6]. Functional aspects of the gastrointestinal tract may be affected by structural integrity. For instance, the rate of fluid absorption in the intestine is affected by the integrity of lateral intercellular spaces which in turn is determined by connective tissue among other things. Glucose absorption is affected by various factors such as rate of diffusion through the intestinal barrier into the intestinal mucosa or even retention within the intestinal wall and enzymatic conversion to lactic acid within the serosa under anaerobic condition. Dietary peroxides could also be broken down extensively in the stomach resulting in other products of lipid oxidation (epoxy ketone of fatty acid) with the later release of oxidized fatty acid from the stomach to the intestine [7].

Palm oil is widely used in domestic cuisines in Nigeria and other African countries and also for the preparation of a number of local products, for example, candles, soaps detergents, cosmetics, and many others, by small and even large industries. It is the most abused oil used in domestic cooking in Nigeria where it is used repetitively to fry bean-cake (*Akara*), plantain, yams, and other food items. This continual heating of the fresh oil usually causes the oil to become thermally oxidized. Studies have shown that thermally oxidized palm oil is injurious to tissues [3, 4]. The literature on the effects of ingestion of thermoxidized palm oil diets on the small intestine is scanty but epidemiologic studies implicated dietary fats as a major risk factor for malignant transformation of the gut in humans [8]. We have demonstrated that chronic ingestion of oxidized palm oil diet causes an increase in basal tone of ileum and enhances intestinal motility and transit in the rat [5]. Since damage to tissues may affect their functions, this study was therefore designed to investigate the effect of palm oil diets on absorption of glucose and fluid transport in the face of these oxidative insults, particularly, from thermoxidized palm oil.

## 2. Materials and Methods

### 2.1. Experimental Animals

Thirty albino Wistar rats weighing 70–100 g were obtained from the animal house of the Department of Physiology of the College of Medical Sciences, University of Calabar, Nigeria. Approval for use of the animals was obtained from the College Ethical Committee on the use of experimental animals. The rats were randomly placed into 3 groups of 10 rats each. The first group, control (C), was fed on normal rat chow. The second group was fed fresh palm oil (FPO) consisting of 15% wt/wt, and the third group was fed diet containing thermoxidized palm oil (TPO) 15% wt/wt. The feeding period lasted for 14 weeks. The animals had free access to water and they were housed in stainless steel cages at room temperature of 28 ± 2°C. 

### 2.2. Formulation of Palm Oil Diet

Twelve litres of fresh palm oil was purchased from a local market in Calabar, Nigeria. It was certified fresh by virtue of its low oxidation status or level of rancidity [9]. The fresh palm oil was divided into 2 equal parts. The first part remained as fresh while the other part was thermally oxidized using the method described previously in [10]. Thermally oxidized palm oil was obtained by heating the fresh palm oil at 150°C in a stainless steel pot intermittently for 5 rounds with each round lasting for 20 minutes. The oil was allowed to cool for 5 hours after each round of heating. The two test diets were formulated by mixing 15% (wt/wt) of each oil with 85% commercial rat feed. 

### 2.3. Measurement of Fluid and Glucose Transport

**Figure 1:**
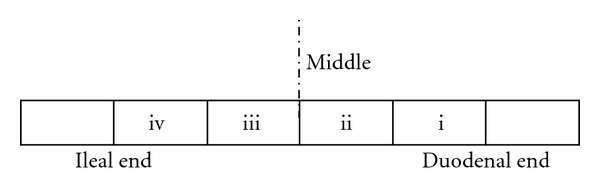


The everted intestinal sac technique described by Wilson and Wiseman [11] used by Barry et al. [12] and modified by Adeniyi and Oloowookurun [13] was used in this study in the measurement of fluid and glucose transport. The animals were rendered unconscious by stunning, and their abdomens quickly cut open to obtain the intestinal segments. Four segments (i, ii, iii, and iv) each 10 cm long were cut out as shown in Figure 1 for sac making. 

The sac was made by tying the distal end of the segment with a dry thread having a standard length, everting the sac before filling it with 1ml Krebs bicarbonate solution (serosal fluid), and tying the other end with a similar thread. Standard Krebs bicarbonate solution that was the mucosa fluid (40 ml) was put in incubating flasks labeled i, ii, iii, and iv, respectively, and each flask was aerated using a 95% O_2_, 5% CO_2_ gas mixture in a Gallenkamp Shaker bath for 30 minutes. The sacs were then immersed in the aerated fluid and aerated further for 2 minutes after which there were incubated for other 28 minutes. After incubation, the sacs were blotted and weighed.

In all, the following weighing was measured: W_1_ = weight of dish + 2 ligatures; W_2_ = weight of dish + empty sac + 2 ligatures; W_3_ = weight of dish + initial full sac + 2 ligatures; W_4_ = weight of dish + final full sac + 2 ligatures; W_5_ = weight of dish + final empty sac + 2 ligatures. Using the Ames/MBI blood analyzer, glucose reagent kit for glucose, the concentrations of glucose in the Krebs Bicarbonate solution and intestinal segments before and after incubation as well as the concentrations in the segments of the sacs after incubation were determined. The fluid and glucose transfer was expressed as ml/g sac/30 minutes) and mg/g sac/30 minutes), respectively, according to Parsons et al. [14] and as used by Barry et al. [12] and Adeniyi and Oloowookurun [13]. Fluid and glucose transfers were determined as measures of volume transferred by a unit wet weight of intestine for a given period.

The mucosal fluid transfer (MFT), serosal fluid transfer (SFT), and gut fluid uptake (GFU) were determined by using the results of the weighing as follows:

 Initial wet weight (IWW) = W_2_ – W_1_;
 Initial serosal volume (ISV) = W_3 _– W_2_;
 Final serosal volume (FSV) = W_4 _– W_5_;
 Serosal fluid transfer (SFT) = FSV – ISV;
 Gut fluid transfer = W_5 _– W_2_;
 Mucosal fluid transfer (MFT) = SFT + GFU.

MFT, SFT, and GFU were expressed as volume /g sac /30 minutes. The terms used for glucose transfer are mucosal glucose transfer (MGT), serosal glucose transfer (SGT) and gut glucose uptake (GGU). MGT is the amount of glucose that disappeared from the mucosal fluid, while the SGT is the amount of glucose that entered the serosal fluid. GGU is the difference in glucose concentration between mucosal and serosal fluids after incubation. This value includes glucose metabolized and those found in the gut wall at the end of the experiment.

### 2.4. Preparation of Tissues for Microscopic Examination

Tissue preparation for microscopic examination was done according to the method of Drury and Wallington [15]. At the end of 14-week feeding period, rats were anaesthetized by inhalation of chloroform and decapitated. The small intestine was removed and placed in cold normal saline. The small intestine was cut open and its contents emptied and the intestine rinsed in normal saline. Tissue blocks from the small intestine were fixed in 10% neutral formation after which they were dehydrated using alcohol and then cleared in xylene. They were then embedded in paraffin wax and thin sections cut at 5 microns. The sections were then stained with hematoxylin for 15 minutes, differentiated with 1% acid alcohol counter-stained in eosin for 2 minutes and mounted with Dextrene polystyrene xylene (DPX). The sections were then viewed under the microscope and photomicrographs were taken.

### 2.5. Determination of Villus Height and Crypt Depth

The villus height was taken as the distance from the crypt opening to the tip of the villus while the crypt depth was measured from the base of the crypt to the level of the crypt opening [16]. Five villi were selected from segment for each rat in the three groups for microscopic analysis, using a 10 × 4 magnifying lens. Histological images were loaded on a 760 × 570-pixel resolution using a computerized image analysis system composed of a Mitotic digital camera (Sony, Japan), installed on a light microscope (Zeiss, Germany) and attached to a personal computer (Pentium IV, 3 GHz; 4.48 MB RAM). Images were captured and displayed on a high-resolution 14-inch colour monitor. The villi height and crypt depth were measured using the Image Pro Plus 4 image analysis software (Media Cybernetics, Baltimore, MD, USA) and expressed as micrometers.

### 2.6. Statistical Analysis

All data are expressed as mean ± SEM. Analysis of data was done using GraphPad Prism software version 5 (GraphPad Software, San Diego, California, USA), and one-way analysis of variance (ANOVA) was used to compare means followed by post hoc Bonferroni test where *P* values were significant. A *P *value of  .05 was considered significant.

## 3. Results

### 3.1. Fluid Transfer in the Intestine of Control and Palm Oil-Fed Groups

The mean values for serosal fluid transfer for control, FPO, and TPO (ml/g sac/30 minutes) were 0.195 ± 0.004, 0.198 ± 0.005, and 0.182 ± 0.058, respectively, (Table 1). There were no significant differences among the groups. The mucosal fluid transfer (ml/g sac/30 minutes) in the TPO (0.293 ± 0.006) was significantly (*P* <  .001) lower compared to either the FPO (0.559 ± 0.007) or control (0.609 ± 0.009) while that for the FPO-fed rats was not significantly different from that of the control. The gut fluid uptake (GFU) in the TPO (0.195 ± 0.004 ml/g sac/30 minutes) was also significantly (*P* <  .001) lower than control (0.414 ± 0.009 ml/g sac/30 minutes) or FPO (0.360 ± 0.005 ml/g sac/30 minutes), no statistically significant difference was recorded in the GFU between FPO and control.

### 3.2. Glucose Transfer by Intestinal Segments of Different Groups of Rats

The values for glucose concentration before incubation in the mucosal and serosal layers for all the groups are presented in Table 2. There was no significant difference among the groups. The value for mucosal glucose transfer after incubation in the TPO (mg/g sac/30 minutes) was 205.45 ± 3.608 which was not significantly different from that for the FPO (208.25 ± 3.59). The serosal glucose transfer for the TPO (172.35 ± 2.97 mg/g sac/30 minutes) was significantly lower (*P* <  .05) than that for FPO (161.95 ± 4.58 mg/g sac/30 minutes) or control (213 ± 8.84 mg/g sac/30 minutes). The gut glucose uptake for TPO (33.85 ± 2.42 mg/g sac/30 minutes) was significantly (*P* <  .01) lower than that for the FPO (46.30 ± 2.19 mg/g sac/30 minutes) or control (57.45 ± 5.6 mg/g sac/30 minutes), respectively.

### 3.3. Villus Height and Depth in the Intestine of Rat Fed with Palm Oil Diet

**Figure 2:**
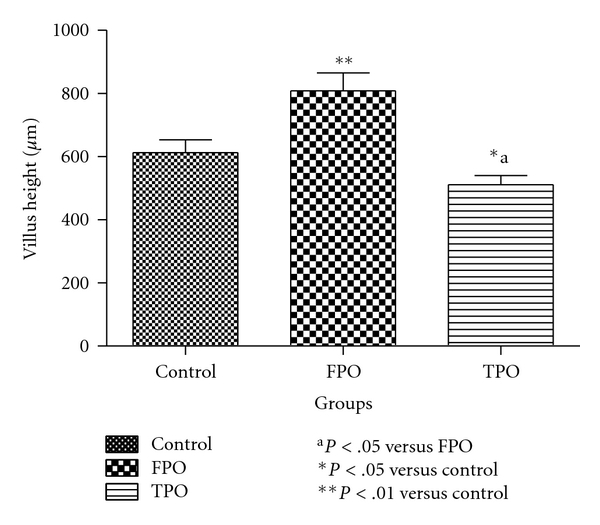
Mean villus height in control, fresh palm oil-, and thermoxidized palm oil-fed rats.

**Figure 3:**
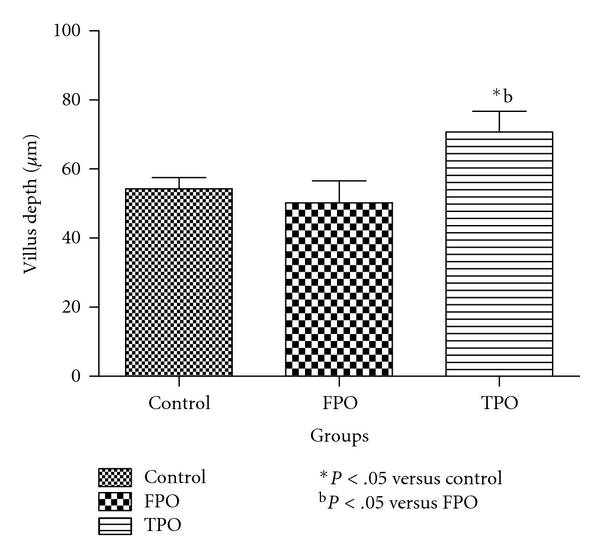
Mean villus depth in control, fresh palm oil-, and thermoxidized palm oil-fed rats.

As shown in Figure 2, the villus height in the control, FPO, and TPO groups was 613 ±   41.00, 808.82 ± 57.00, and 511.56 ± 29.00 *μ*m, respectively, showing a significant (*P* <  .05) reduction in the TPO compared with the control and FPO groups, respectively. The villus height in the FPO group was significantly greater than that in the control group (*P* <  .01). The villus width in control, FPO, and TPO groups were 54.30 ± 3.21 *μ*m, 50.18 ± 6.42 *μ*m, and 70.70 ± 6.01 *μ*m, respectively. The villus depth in the TPO group was significantly higher (*P* <  .05) than that in the control or FPO groups, respectively, (Figure 3). The villus depth in the FPO group was not significantly different from the control.

## 4. Discussion

The results on the study of effect of palm oil diets on intestinal fluid and glucose absorption in rat show that as a result of administration of TPO, animals showed a decrease in fluid and glucose uptakes in comparison with control. Also, villus height in the TPO group was significantly lower than that in control. These results may be attributed to the fat content and quality of palm oil present in the diet. Palm oil in its fresh state has vitamins A and E, carotenoids, and other antioxidants which make it less injurious to the tissues when compared with the thermally oxidized form that lacks these vital complements [17]. Oxidation of palm oil leads to release of free radicals which are devastating to tissues and organs of the body [3, 6, 17–21].

 The damage affecting the height and width of villi in the thermoxidised palm oil-fed rats may be due to the devastating effect of these free radicals. The distortion in villus height and width may invariably be responsible for the malabsorption of fluid and glucose in this group of rats. 

The villus height in the intestine obtained from rats fed on fresh palm oil was higher than that in thermoxidized palm oil fed rats. This may be due to the fact that a variety of antioxidants present in fresh palm oil are capable of protecting the stomach and intestine from necrotizing effect of free radicals as they help increase mucus secretion [22]. A study had shown that arachidonic acid (AA), docosahexaenoic acid (DHA), or low-n6/n3 diet had no effect on villous height, compared with effect of the high-n6/n3 diet whereas the AA and DHA diets were associated with an approximately 15% reduction in villous height in intestine thus demonstrating influence of dietary composition on intestinal function [23]. A previous study had also demonstrated that, in rats fed the high saturated fatty acid diet, there was reduced mean ileal villus height, width, thickness, surface area, cell size, and villus density, as well as reduced mucosal surface area compared with unsaturated fatty acid diet [24]. The noted increase in villus height in FPO group compared with TPO and control could be attributed to accelerated intestinal growth since dietary palmitic acid modulates intestinal growth in rats [25]. Fresh palm oil contains a higher content of palmitic acid than thermally oxidized palm oil. Since the villi are not as distorted as in the fresh palm oil-fed rats, there was improved absorption of glucose and fluid in the FPO group. That the fluid and glucose absorption in the FPO was still lower than that in control may be pointing to the fact that there might still be slight oxidation of fresh palm oil owing to exposure to sunlight which could cause some absorption impairment [26]. This could be so because of auto-oxidation of palm oil [27]. The results from the present study suggest that chronic use of thermally oxidized palm oil may lead to fluid and glucose malabsorption in the small intestine in rats. 

## Figures and Tables

**Table 1:** Fluid transfer in intestine of the rats fed on palm oil diets.

Groups	Body weight range (g)	Serosal fluid transfer (ml/g sac/30 minutes)	Mucosal fluid transfer (ml/g sac/30 minutes)	Gut fluid uptake (ml/g sac/30 minutes)
Control	280–310	0.195 ± 0.004	0.609 ± 0.009	0.414 ± 0.009
FPO	280–320	0.198 ± 0.005^NS^	0.559 ± 0.007^NS^	0.360 + 0.005^NS^
TPO	270–290	0.182 ± 0.058^NS, a^	0.293 ± 0.006^∗∗∗, b^	0.195 ± 0.004^∗∗∗, b^

Each value represents mean + SEM, for 20 sacs in 5 rats; NS: not significant versus control;

***P <  .001 versus control; ^a^not significant versus FPO; ^b^ *P* <  .001 versus FPO.

**Table 2:** Glucose transfer by intestinal segments of rats fed on palm oil diets.

Groups	Body weight range (g)	Glucose conc. Before incubation (mg/g sac/30 minutes)		Glucose transfer after incubation		Gut glucose uptake
Mucosal	Serosal	Mucosal	Serosal
Control	280–310	122.40 ± 1.82	122.40 ± 1.82	271.30 ± 10.47	213.85 ± 8.84	57.45 ± 5.60
FPO	280–320	120.00 ± 1.00	120.00 ± 1.00	208.25 ± 3.58	161.95 ± 4.58	46.30 ± 2.19
TPO	270–290	122.40 ± 1.12	122.40 ± 1.12	205.45 ± 3.61	172.35 ± 2.97*	33.85 ± 2.40**

Each value represents mean + SEM, for 20 sacs in 5 rats, *P <  .05, **P <  .01 versus FPO.
